# IMMUNE ZONES IN LEPROSY

**DOI:** 10.4103/0019-5154.55626

**Published:** 2009

**Authors:** T S Rajashekar, Gurcharan Singh, L Chandra Naik

**Affiliations:** *From the Department of Dermatology, Sri Devaraj Urs Medical College, Tamaka, Kolar - 563 101, Karnataka, India.*

**Keywords:** *High local temperature*, *immune zones*, *relatively immune*

## Abstract

Leprosy affects mainly those areas of skin which have a relatively lower temperature and are more exposed to trauma. Certain zones like scalp, palms and soles, genitalia, groins, axillae, eyelids, transverse band of skin over lumboscaral area, midline of back and perineum have been described to be immune to the development of lesions in leprosy. But clinical, histological and bacteriological evidence of involvement of these so called immune zones though infrequent have been documented. Hence, these immune zones should be termed as relatively immune, rather than absolutely immune zones of leprosy.

Leprosy affects mainly those areas of the skin, which have a relatively lower temperature and are more exposed to trauma. Thus, leprosy lesions are found commonly over the face, knees, elbows, gluteal region, dorsal aspects of the extremities, and trunk.[1,2] Yet, certain zones such as scalp, palms and soles, genitalia, groins, axillae, eyelids, transverse band of skin over lumboscaral area, and midline of back and perineum have been described to be immune to the development of lesions in leprosy.[3–5] The reason for sparing of these zones has been attributed to the relatively high local temperature.[2,3,6] However, clinical, histological, and bacteriological evidence of involvement of these so-called immune zones though infrequent has been documented and briefly reviewed in the succeeding text.

## Scalp as an Immune Zone in Leprosy

Scalp is considered to be one of the immune zones in leprosy in addition to other sites mentioned.[7–14] However, Muir (1938) states that “lesions on the scalp are quite common though the denseness of the hair and the covering provided by it renders the lesions on the scalp less conspicuous”.[14]

Scalp involvement in leprosy can be classified into:

1. Leprotic alopecia;
2. Involvement of the bald area of the scalp;
3. Extension of anesthesia from neighboring lesion;
4. Apparently normal skin showing AFB in histopathological sections or slit skin smear examination;
5. Involvement of hairy area of scalp.[7]

Oteig and Pinegro (1960) classified leprotic alopecia into,

1. Diffuse alopecia;
2. Regional alopecia localized to temple;
3. Circumscribed alopecia;
4. Mitsuda's type;
5. Wig-type.[7,15]

Leprotic alopecia is more common in the temporal area of scalp, but the area overlying the course of the temporal artery is spared.[16–18] Leprotic alopecia, which is seen in Japanese patients suffering from lepromatous leprosy, has been well illustrated by Mitsuda.[18] Cochrane, referring to the rarity of this condition observed that in certain races particularly the Mongolian and occasionally European, a leprous alopecia is sometimes seen. Scalp involvement is also rare in African patients but never as far as known among the Indians.[3,8,10]

Involvement of hairy scalp is considered to be very rare, the scanty reports of scalp involvement in leprosy have been mostly on the bald areas of scalp.[9,19,20] Hairy scalp has higher skin temperature than the other parts by approximately 5°C.[2,6] It is well known that *M. leprae* has more predilection for cooler parts of the body, hence hairy scalp involvement will naturally be rare.[21,22]

Two cases with tuberculoid lesion on the hairy occipital area of the scalp, well inside the hairline were reported.[12,20] Though rare, the hairy scalp can be involved in the borderline tuberculoid leprosy, and the hair growth may appear normal.[11,23,24] Fleury *et al.* and Malaviya *et al.* reported plaques and nodules over the scalp in lepromatous leprosy patients.[9,10]

## Involvement of palms and soles

**Figure 1:**
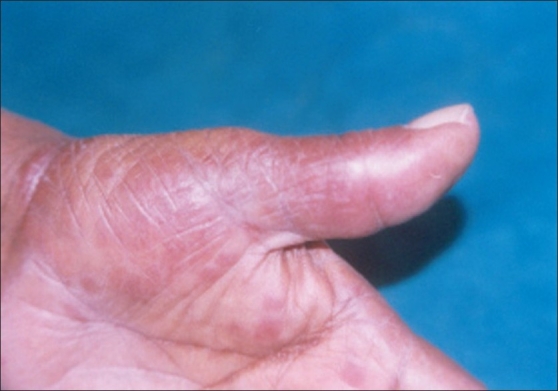
Erythematous plaque over left palm

Leprosy affects mainly those areas of skin, which have relatively lower temperature and are more exposed to trauma.[1,21,22] Palms and soles are cooler than the rest of the body, more prone to trauma and have rich nerve supply and are thus expected to be involved more frequently. However, they are considered to be rarely involved [Figure 1].[1,3]

These areas differ from other superficial areas of the skin in two ways:

1. The epidermis is thickest on the palms and soles, measuring approximately 1.5mm which is slightly thicker than that of other superficial skin areas and hence comparatively warmer.[1,5]
2. There is a fairly good amount of fibrofatty tissue which ensures an insulating property and hence a high nerve bed temperature.[1,25,26]

Temperature of the nerve bed is directly related to the depth of the tissues. Thus, there is every likelihood of nerve bed temperature of the palmo-planter regions higher than that of other superficial skin regions, on account of the above said reasons, which renders the palmo-planter localization of *M. leprae*, quite less likely.[26,27]

Lesions over the palms and soles have been reported by several workers in all types of the disease.[28] Rajendran reported three cases of tuberculoid leprosy with palmo-planter lesions.[26] Aggarwal *et al.* reported a case of tuberculoid leprosy which presented with a primary annular lesion on the sole of the left foot.[5] Sharma reported 3 histologically confirmed tuberculoid cases involving the sole of the foot.[29]

Chattopadhyay *et al.* reported a case of borderline tuberculoid leprosy in reaction with lesions over the uncommon sites like palms and soles.[30] Grover *et al.* reported an uncommon case of borderline tuberculoid leprosy which had primary hyper-pigmented palmar lesion.[31] Pavithran reported primary skin lesions of leprosy on the palm and sole in two patients, one of them having nodular lepromatous leprosy and the other having borderline tuberculoid type of the disease.[32] Baslas reported a case of histoid lepromatous leprosy who had palmar involvement.[33]

Hopkins *et al.* screened 245 leprosy patients for lesions over certain anatomical locations and found palmar involvement in 17 (6.9%) and planter involvement in 13 (5.9%) cases.[34] Indira *et al.* carried out a study to assess the frequency of lesions over palms and soles. Of the 280 leprosy patients screened, 28 (10%) showed lesions over the palms and/or soles, 12 (42.8%) had only palmer lesions, 6 (21.4%) had only planter lesions, and 10 (35.7%) had both palmer and planter lesions. Palmoplanter lesions were found in BT, BL and LL types of diseases.[1]

Palmoplanter lesions have also been described in leprosy patients in literature.[1,30]

## Involvement of external genitalia and scrotum

**Figure 2:**
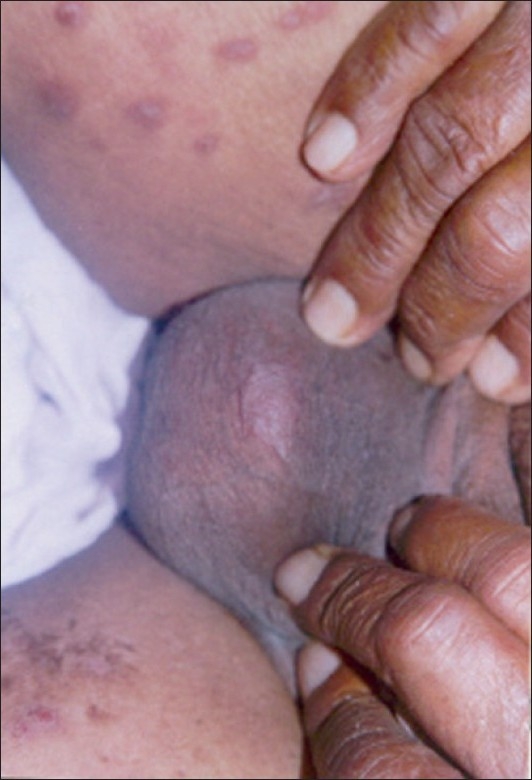
Infiltrated plaques and nodules over scrotum and thighs

The male external genitalia was considered to be immune to the occurrence of the leprosy lesions,[35] in spite of the lower temperature of the scrotum and testicles, which favors the growth of *M. leprae*.[36] However, clinical involvement of the external genitalia and scrotal skin has been reported by several workers in all types of the disease spectrum [Figure 2].

Primary involvement of scrotum has been reported in indeterminate[37] and tuberculoid type of leprosy.[38] Single, well-defined anaesthetic plaque was reported over scrotum,[39] preputial skin[40], and over penoscrotal fold[41] in tuberculoid Hansen's disease.

Two different cases with borderline leprosy with a progressive, asymptomatic, hypopigmented, hypoaesthetic plaque situated over the anterior surface of the scrotum, sparing the penile skin completely,[42] and with a primary involvement of the scrotum[43] were reported. Arora *et al.* found cutaneous lesions of male genitalia in 2.9% of all cases examined. Most of them were of borderline type.[43]

Clinical involvement of scrotal skin in lepromatous leprosy[44] and genital nodules and testicular hydrocele in a case of relapsed lepromatous leprosy[36] were reported. Lesions over the genitalia including glands and scrotum were reported in a case of histoid leprosy.[45,46]

Six leprosy patients in the Ridley-Jopling spectrum of BT-BL showing lesions on the penis and scrotum were reported.[47]

Histopathological and bacteriological involvement of the scrotal skin in lepromatous leprosy has been documented by Pandya *et al.* and Ramu *et al.*[48,49] In a study by Ramu *et al.*, scrotal biopsies were obtained from 38 cases of LL who had clinically subsided lesions with negative skin smears. Twenty-six (68.4%) of these cases revealed bacilli in the dartos muscle. None except one showed a specific lesion in the dartos. Bacilli obtained from two out of seven cases multiplied in the mouse foot-pad.[49]

Bhushan Kumar *et al.* observed 6.6% cases with genital lesions of 467 male patients examined in one study. They were seen most frequently in LL (25.8%) followed by BL (13.3%) and BT (1.4%) leprosy.[50]

Scrotal skin has been reported to be relatively cooler than the core temperature for effective spermatogenesis.[37,41] However, due to the use of heavy undergarments, it is likely that the temperature of the scrotal skin may remain elevated.[50]

Thus, after various studies and reports, it is not uncommon to find cutaneous lesions of leprosy on male genitalia and scrotum. It seems that paucity in literature of external genitalia and scrotum is due to either to effort by the patient to conceal it or reluctance of doctor or health worker in exposing the patient.[36,41,43]

## Involvement of other immune zones in leprosy

The groin, axillae, perineum, eyelids, midline of back and the transverse band of skin over the lumbosacral area have been described as “immune-zones” with respect to the development of cutaneous lesions of leprosy.[3,42]

### Involvement of groin

Sahni *et al.*, studied twenty untreated BL and LL cases. They observed groin involvement clinically in five cases, skin-smear positivity in three, while all showed histological changes.[51] Bedi *et al.*, observed histological involvement of groin in 10 out of 20 lepromatous leprosy patients.[52]

### Involvement of axilla

Anish demonstrated higher temperature of the axilla as compared to that of the forearm.[6] The cutaneous lesions of borderline leprosy were found in uncommon sites like axillae and palms and soles.[23] Jayakumar *et al.* reported a case of advanced lepromatous leprosy having obvious lesions over the scalp and lepromatous infiltration of the axillae and groins.[14]

### Midline of back

Hastings *et al*. reported that even regional temperature differences noted in the cooler lateral back, versus the warmer midline, influenced the bacterial invasion.[53]

**Figure 3:**
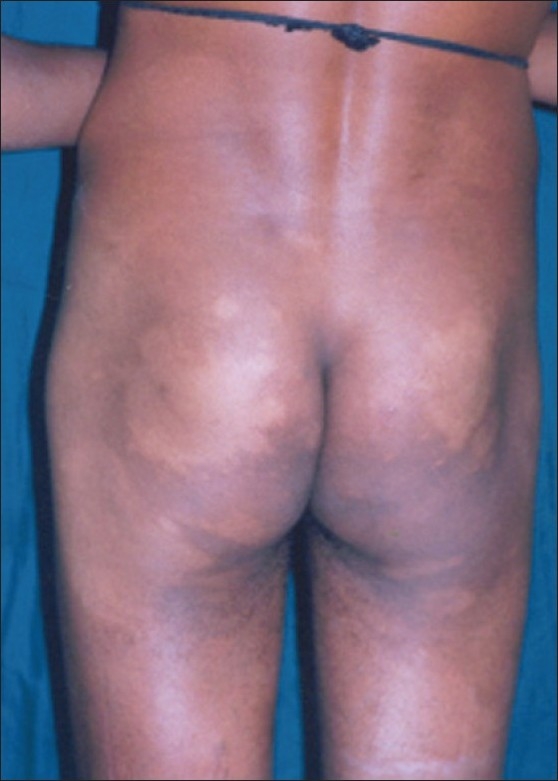
Hypopigmented patches over midline back and buttocks

The clinical, bacteriological and histopathological features were studied in 20 cases of leprosy (10 LL and 10 BL) from the so-called immune zones that are axilla, groin and midline of back. In these zones the clinical lesions were noted in 40% of the cases (7 LL and 1 BL), AFB were detected in the smears of 45% cases (8 LL and 1 BL) and histopathological evidence of the disease was observed in almost all the sites studied(100%). Midline of back was the commonly affected site [Figure 3], followed by axilla and groin.[51]

### Eyelids, perineum, and narrow transverse band of skin over the lumbosacral region

Eyelids, perineum, and narrow transverse band of skin over the lumbosacral region on the back have has also been described to be immune to the development of the lesions in leprosy[3] because of the relatively high local temperature.[2]

### Leprous involvement of clinically normal appearing skin

No skin area is immune to the invasion of *M. leprae*, as studies have documented bacteriological and histological evidence of disease process in clinically uninvolved skin in leprosy patients.[54,55]

Skin biopsies of clinically normal skin of the scalp, axillary, and groin regions in 20 lepromatous leprosy patients revealed significant histopathological findings in upto 25% of the patients.[52]

## Conclusion

Therefore, it is not uncommon to find cutaneous lesions of leprosy on unusual sites such as scalp, palms and soles, genitalia, groins, axillae, eyelids, transverse band of skin over lumboscaral area, midline of back and perineum which should be termed as relatively immune, rather than absolutely immune zones of leprosy.
